# Serum Leptin as a Marker for Severity of Endometriosis

**DOI:** 10.1155/2020/6290693

**Published:** 2020-09-07

**Authors:** Shatha Sami Hussein, Fatin Shallal Farhan, Alaa Ibrahim Ali

**Affiliations:** Al-Mustansiriyah University, College of Medicine, Department of Obstetrics & Gynecology, Baghdad, Iraq

## Abstract

**Background:**

Endometriosis a disease of theories, and one of the important causes of chronic pelvic pain, dysmenorrhea, dyspareunia, and subfertility. Surgery is the mainstay step for the diagnosis; noninvasive test is the goal in the future. *Aim of Study*. To test the role of serum leptin in determination of severity of endometriosis. *Study Design*. A cross-sectional study done in Al-Yarmouk Teaching Hospital from 1st of January 2018 to 1st of January 2019.

**Methods:**

60 BMI-matched patients were involved in the study. A study group of 30 patients were operated either by laparoscopy or laparotomy for many reasons diagnosed as endometriosis by histopathology, and 30 normal women as a control group underwent elective surgery. Blood sample was taken from all patients in the theater room when laparoscopy finding went with endometriosis, and classifying according to surgical staging of endometriosis, the level of serum leptin was measured by ELISA using Human LEP (Leptin) ELISA Kit. The recording of finding of laparoscopy after conforming of diagnosis by histopathology was compared with the result of serum leptin.

**Result:**

The result shows no significant difference between the two groups regarding parity and age; however, the level of serum leptin was significantly high in the endometriosis group than in the control group. The *P* value was less than 0.05. Also, the result shows no significant differences between serum leptin in both groups according to the symptom but there was a significant difference with surgical staging. The mean of the level of serum leptin in stage 1 was 214.8, while it was 340.3 in stage 4.

**Conclusion:**

Serum leptin can be used as a marker of severity of endometriosis.

## 1. Introduction

Endometriosis is an enigmatic disease associated with serious morbidity and change in quality of life among childbearing-age females. Early diagnosis and focus management of the disease were a big challenge for both gynecologists and patients. It is defined as endometrial tissue (stroma and gland) present outside the uterus [1]. Determination of the endometriosis incidence is challenging because some women do not show symptoms; prevalence was estimated to be between 10 and 15 percent of women [2]. Also, with Mullerian abnormality, incidence increased up to 40%. The endometrial tissue may present in any site according to the process of its development and progress such as ovary, peritoneum, bladder, vulva, and scar of operation [3] or in rare sites such as brain tissue [4]. These ectopic tissues remain under the effect of ovarian hormones which cause cyclical change of growth and shedding that lead to wide variation of symptoms due to fibrosis and adhesion formation and infiltration [5].

More than 100 years ago when endometriosis was first described, its nature, progression, and the way by which it was related to infertility and occurrence of pelvic pain remain unclear [6].

The etiology of endometriosis appears to be of multiple theories, including hematological metastasis, immunological changes, abnormal proliferation of the cell and apoptosis, endocrine abnormality, and genetic predisposition (stem cell theory) [7].

There is no specific test for diagnosis of endometriosis. While many markers were evaluated in many research studies for the noninvasive diagnosis of disease, none were revealed to be of great benefit. CA125 concentrations show a high level in patients with endometriosis but they are not specific [8].

Leptin is a 167 amino acid protein with a 21 amino acid signal peptide and a product of ob gene. Leptin is present in the plasma in two forms, free or bound to leptin-binding proteins. This hormone had a role in basal metabolism, reproduction, and food intake. Leptin also had immune-regulatory, proinflammatory, and neoangiogenesis functions, so it may play a role in pathogenesis of endometriosis [9].

## 2. Patient and Methods

A cross-sectional study was performed in Al-Yarmouk Teaching Hospital (tertiary hospital in Baghdad) from 1^st^ of January 2018 to 1^st^ of January 2019.

60 BMI-matched patients were involved in the study. A study group of 30 patients were operated (laparoscopy or laparotomy for ovarian cyst, chronic pelvic pain, and painful lump at the site of previous scar) and all were diagnosed as endometriosis by histopathology. 30 normal women underwent laparoscopy for other gynecological causes such as sterilization, diagnostic laparoscopy for infertility, and chronic pelvic pain and no endometriosis) as the control group. Verbal consent was obtained from all patients involved in the study.

Inclusion criteria included all patients that had pelvic pain, infertility, dysmenorrhea, dyspareunia, and suspected to be a case of endometriosis and underwent laparoscopy for diagnosis.

Exclusion criteria included pregnant patients, haemodynamically not stable patients, obese patients, and patients with medical disease. Detailed history includes the chief complaint signs and symptoms and medical, surgical, drug, and menstrual history. Blood sample was taken from patients in the theater room when laparoscopy finding went with endometriosis and it was confirmed lately by histopathological examination, and the level of serum leptin was measured by ELISA by using Human Leptin ProQuantum Immunoassay kit and recording of finding of laparoscopy and the severity was classified according to the revised American Society for Reproductive Medicine Scoring system:

- Stage I: minimal, isolated implants, and no significant adhesions.
- Stage II: mild, superficial implants <5 cm in aggregate, scattered on the peritoneum and ovaries, and no significant adhesions.
- Stage III: moderate multiple implants, both superficial and deeply invasive. Peritubal and periovarian adhesions present.
- Stage IV: severe, multiple superficial and deep implants. Presence of large ovarian endometriomas. Filmy and dense adhesions are usually present [10].

Also, the result of serum leptin was then compared between the study groups.

## 3. Statistical Analysis

Data analysis was performed by using Statistical Packages for Social Sciences-version 25 (SPSS-25).

The relation between differences of variable means was tested using Student's-*t*-test, and ANOVA test was used for difference among more than two independent means. The significance of difference of different percentages (qualitative data) was tested using Pearson's chi-square test (*χ*^2^-test) with application of Yate's correction or Fisher's exact test whenever applicable. Statistical significance was considered whenever the *P* value was equal to or less than 0.05.

## 4. Results

Table 1 shows the demographic differences between the two groups, and it shows significant difference regarding parity and age because most of the patients with endometriosis present with infertility and low parity and more prevalence at 30–40 years.

**Figure 1:**
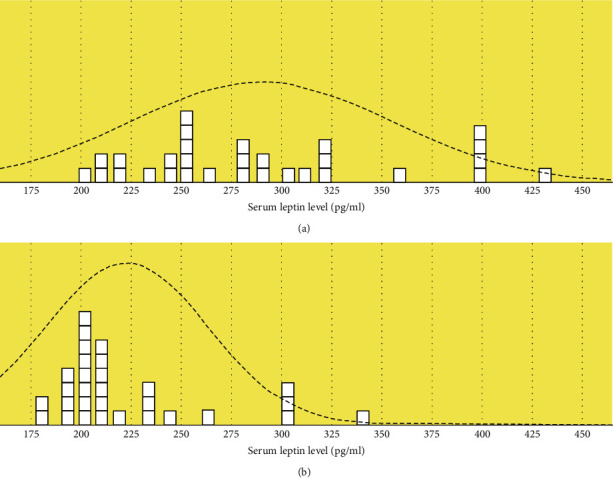
Serum leptin curve in both groups. (a) Endometriosis. (b) Controls.

Serum leptin is significantly high in the endometriotic group than in the control group. The *P* value is less than 0.05 as shown in Table 2 and Figure 1.

Table 3 shows no significant differences between serum leptin in both groups according to symptoms, but there was a significant difference with surgical staging with a *P* value of 0.0001.

**Figure 2:**
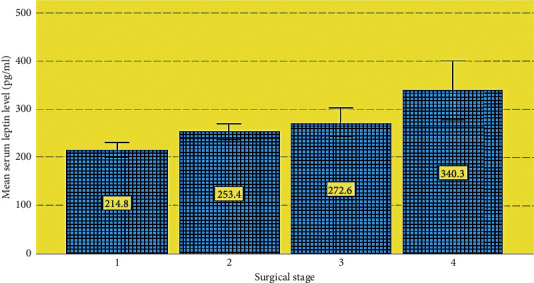
Serum leptin level in relation to surgical staging.

Figure 2 shows that the mean level of serum leptin was 214.8 in stage 1 and increase indirect relation of increment to be 340.3 in stage 4.

## 5. Discussion

Endometriosis is a progressive, chronic condition. It has been recently reported that serum concentrations of leptin play a role in reproduction. On the basis of these findings, studies evaluating the serum leptin level try to show the relationship between the serum leptin level and endometriosis and if it had a role in the pathogenesis of this disease, the relationship between the serum leptin and endometriosis severity is still controversial; several studies revealed there was no relation while others have shown a positive correlation with more severe forms of endometriosis.

In Zendron et al.'s [11] study, 25 women were enrolled , 10 as control and 15 patients undergoing surgery for adnexal mass, and the leptin levels in both serum and peritoneal fluid (PF) and the protein expression in different peritoneal implants were studied. The study revealed that the level of leptin was higher in the endometriosis group but had no clear role in the progression of disease. The result matches our study in relation to increase in the serum leptin level significantly in the patient with endometriosis, but our study revealed that there was a direct significant relation with the severity.

In [12], 13 women with variable stages of endometriosis and 15 patients as control were enrolled. The serum leptin level and the result revealed statically significant increase in the endometriotic group which has a similar match with our study which indicates the possible role in the pathogenesis of endometriosis.

Leptin has recently been suggested to be involved in unexpected functions, specifically the process of angiogenesis and immune response which are one of the most important factors involved in the pathogenesis of endometriosis.

Viganò et al.'s [13] study involved 67 women divided into those proved to have endometriosis and remaining as control. The result revealed no significant difference so it is not a marker for diagnosis of endometriosis or does not detect its severity. This result disagrees with our study.

Osman et al. [14] studied the role of leptin and some other antioxidants in infertile women, with endometriosis blood sample collected from 38 patients, about two thirds of them being in the study group and the others in the control group. It was revealed that there was no important difference in serum leptin concentrations between the studied groups. Again, this study disagrees with our study.

Wertel et al. [15] compared the level of serum and peritoneal level of leptin in different stages of endometriosis in two study groups, fertile and nonfertile, and the study revealed that higher level PF leptin concentration was observed in patients with stages III and IV of endometriosis than in those with the minimal stage of the disease, a similar result from our study in serum leptin level.

Leptin may be used with other markers as combination to predict the severity of endometriosis.

## Figures and Tables

**Table 1:** Demographic characteristic features of both groups.

		Endometriosis (30)	Controls (30)	*P* value
Age (years)	<20	0 (0.0)	2 (6.7)	0.031^*∗*^
	20–24	0 (0.0)	7 (23.3)	
	25–29	8 (26.7)	6 (20.0)	
	30–34	14 (46.7)	10 (33.3)	
	≥35	8 (26.7)	5 (16.7)	
	Mean ± SD (range)	32.3 ± 4.4 (25–42)	29.0 ± 6.6 (16–42)	0.029^#^

Parity	0	8 (26.7)	1 (3.3)	0.016^*∗*^
	1–4	20 (66.7)	22 (73.3)	
	≥5	2 (6.7)	7 (23.3)	
	Mean ± SD (range)	1.7 ± 1.5 (0–6)	3.0 ± 1.6 (0–6)	0.002^#^

^*∗*^Significant difference between proportions using Pearson's chi-square test at 0.05 level. ^#^Significant difference between two independent means using Student's-*t*-test at 0.05 level.

**Table 2:** Relation between serum leptin and both groups.

		Endometriosis (30)	Controls (30)	*P* value
Serum leptin level (pg/ml)	150	0 (0.0)	8 (26.7)	0.0001^*∗*^
	200	8 (26.7)	17 (56.7)	
	250	11 (36.7)	1 (3.3)	
	300	5 (16.7)	4 (13.3)	
	350	2 (6.7)	0 (0.0)	
	400	4 (13.3)	0 (0.0)	

	Mean ± SD (range)	289.73 ± 65.08 (201.245–431.479)	222.66 ± 40.40 (180.492–340.450)	0.0001^#^

^*∗*^Significant difference between proportions using Pearson's chi-square test at 0.05 level. ^#^Significant difference between two independent means using Student's-*t*-test at 0.05 level.

**Table 3:** Serum leptin level according to symptoms and surgical staging in group 1.

		Serum leptin level in endometriosis group	
		No	Mean ± SD
Symptoms	Infertility	14	286.3 ± 60.5
	Pelvic pain	12	279.2 ± 61.2
	Others	4	333.3 ± 91.2
	*P* value		0.353
Surgical stage	Stage 1	5	214.8 ± 15.5
	Stage 2	4	253.4 ± 17.8
	Stage 3	8	272.6 ± 31.5
	Stage 4	13	340.3 ± 61.2
	*P* value		0.0001^*∗*^

^*∗*^Significant difference among more than two independent means using the ANOVA test at 0.05 level.

## Data Availability

The patient data used to support the findings of this study are currently under embargo while the research findings are commercialized. Requests for data 6/12 months after publication of this article will be considered by the corresponding author.
